# Frequency and predictors of emergency department visits among the oldest old in Finland: the Vitality 90+ Study

**DOI:** 10.1186/s12913-025-12923-2

**Published:** 2025-06-05

**Authors:** Sherin Billy Abraham, Jutta Pulkki, Mari Aaltonen, Esa Jämsen, Jani Raitanen, Linda Enroth

**Affiliations:** 1 Faculty of Social Sciences, Unit of Health Sciences, Tampere University, Tampere, Finland; 2 Unit of Services (Older People Services), Finnish Institute for Health and Welfare, Helsinki, Finland; 3 Faculty of Medicine (Clinicum), University of Helsinki, Helsinki, Finland; 4 Department of Geriatrics, Helsinki University Hospital, Helsinki, Finland; 5 The UKK Institute for Health Promotion Research, Tampere, Finland; 6 Faculty of Social Sciences, Unit of Health Sciences and Gerontology Research Center, Tampere University, Tampere, Finland

**Keywords:** Acute care, Emergency medical services, Frequent ED use, Longevity, Round-the-clock care, Community-dwelling

## Abstract

**Background:**

Emergency department (ED) visits increase with age. However, knowledge about the frequency and drivers of ED visits among the oldest old (90 years and above) is scarce, and neither is it known whether patterns differ between those living at home and in round-the-clock care. As understanding of ED use and the factors influencing ED visits is crucial for the functioning of health care systems, this study examined the frequency and predictors of ED visits in a 90+ population living at home and in round-the-clock care.

**Methods:**

Data from the Vitality 90+ survey, a population-based study with 1561 respondents in 2014 in Tampere, Finland, was combined with national register data on ED use and mortality until the end of 2017. Predictors of the first ED visit were examined using Cox regression models (4-year cumulative hazard) and the frequency and predictors of 1–3 and ≥ 4 ED visits in one year of follow-up with multinomial logistic regression models.

**Results:**

Over the four-year study period, 79% of the participants had at least one ED visit. Those living at home had higher cumulative hazards of ED visits and were more often frequent ED users (≥ 4 ED visits) than those living in round-the-clock care. Not receiving home care, multimorbidity, poor subjective health and wellbeing, and limitations in functioning increased the risk of ED visits among home dwellers, while having dementia, limitations in functioning, impaired sensory functions, and less frequent social contacts decreased the risk among round-the-clock care residents. In both groups, the predictors of ED visits were similar in one- and four-year follow-ups.

**Conclusions:**

The frequency and predictors of ED visits greatly differ between those living at home and in round-the-clock care. Since most ED visits occurred among those living at home and having poor health but not receiving formal home care, improving the continuity of care and the coverage of home care services could help to curb the increase in ED visits among the fast-growing oldest old population.

**Supplementary Information:**

The online version contains supplementary material available at 10.1186/s12913-025-12923-2.

## Background

People worldwide are living longer than before. The oldest old population (90 years and above) has grown at an unprecedented rate, climbing from 8.5 million in 2000 to 21.3 million in 2020 [1]. A similar trend is observed in Finland, where the number of individuals aged 90 and over has more than doubled during the same period (from 22,637 to 56,847). The age group now accounts for about 1% of the country’s population [2].

Multimorbidity, usually defined as ≥ 2 co-existing conditions in an individual [3], disabilities, and care needs increase with age, with major implications for health and social care services. One of the effects is the higher emergency department (ED) visit rate among older adults. In a high-volume collaborative ED with a catchment area of more than one million people in Finland, the number of ED visits was higher among those aged 90 or over (1007/1000 person years) compared to those under 80 years (233/1000 person years) between 2015−2016 [4], indicating an increasing frequency of ED use even in very old age. Health problems such as reduced functioning and multimorbidity have been identified as the most important causes for ED visits [5, 6]. The literature also shows that complex medical problems such as cardiac, respiratory, and cerebrovascular-related conditions, fall-related injuries, and physical and cognitive impairments, are associated with ED visits [7, 8].

Sociodemographic factors have been found to contribute to ED visits among older adults. Crimmins et al. found different morbidity profiles for women and men [9]. Women are more susceptible to ED visits than men as they live longer, more often live alone, and have more disabling health problems [10]. However, the results on gender differences in ED visits are conflicting [11]. High socioeconomic status has been shown to be associated with better health, even among the oldest old individuals [12], as well as with higher social support and better financial situation [13, 14]. Thus, those with lower socioeconomic status may have higher needs for health care services, including ED, but fewer opportunities to seek help for medical conditions. Living arrangements are often linked with the availability of formal or informal care. It has been suggested that older adults living alone or in institutional long-term care use more ED services than those living with a family or spouse [13, 15]. However, research on the role of living arrangements and receipt of formal or informal care in relation to ED visits is limited [5].

There is no standard definition for frequent ED use, but the commonly used cutoff is four or more visits a year. Using this cutoff, Dufour et al. observed that 6.6% of older adults were frequent ED users and accounted for 38% of all ED visits in a one-year follow-up [16]. Further, studies have reported that frequent ED use is positively associated with higher age, low income, presence of chronic diseases, a higher number of past ED visits and hospital admissions, and a higher number of medications. Dementia and better access to and continuity of health care are associated with less frequent ED use [14, 16]. Studies using the same cut-off for frequent ED use report worse patient outcomes such as hospitalization, re-visits, unmet health care needs, multimorbidity, and in-hospital mortality [13, 16, 17].

The vast majority of studies into ED visits among older people are conducted among the general older population aged 65 or over. In recent years, however, research has drawn attention to the fast-growing and vulnerable oldest old age group, which differs considerably from the younger older adult population in terms of mortality rate, health status, and care needs [18]. This study examines the frequency and predictors of ED visits among the oldest old population in the city of Tampere, Finland. As the circumstances and needs of individuals living at home and in round-the-clock care vary considerably [19, 20], we wanted to explore the similarities and differences in ED visits between these two groups. Our research questions are: (1) What is the frequency of ED visits and what are the predictors of the first ED visit among the oldest old individuals living at home and in round-the-clock care from 2014 to 2017? (2) What is the frequency and what are the predictors of 1−3 and ≥ 4 ED visits in one year of follow-up (2014−2015) among the oldest old individuals living at home and in round-the-clock care? A better understanding of the predictors of ED use among the oldest old may help in planning effective services for this vulnerable population and easing the burden on already congested emergency services.

## Methods

The Vitality 90+ Study [21], initiated in 1995, is an ongoing study of the oldest old individuals in Tampere, Finland, a city with a population of around 235,000 (2020). The data for the current study were collected by mailed surveys from all individuals aged 90 or over living at home and in round-the-clock care. These data were from the 2014 survey wave, which yielded 1637 participants with an 80% response rate. Proxy respondents (19%) were used for those unable to respond for themselves.

The survey data were linked with national register data on ED visits to specialized (Care Register for Health Care, Finnish Institute for Health and Welfare (THL)) and primary health care (Register for Primary Health Care Visits, THL) and date of death (Causes of Death register, Statistics Finland) through unique personal identity codes that are given to all residents in Finland. The analytical sample included 1561 participants who consented to have their survey data linked with the register data. The baseline for the survey was 17th January 2014. Entry to ED and mortality were followed for three years and eleven months (until 31st December 2017).

### Outcome variable

ED provides round-the-clock acute health care for patients whose health could be compromised due to delay in health care [22]. Information on ED visits covers both primary and specialized care in the area. First, we examined time to the first ED visit (cumulative hazard) during four years of follow-up (17th January 2014–31st December 2017). Second, we examined the number of ED visits for one year (17th January 2014–16th January 2015) to quantify the frequency of ED visits. ED visits were categorized as 0, 1−3 and ≥ 4 visits, of which the latter was defined as frequent ED use.

### Explanatory variables

Earlier research [13–15] has demonstrated the multifaceted associations of health and functioning, social environment, and availability of home help with ED visits. Therefore, we chose to explore several possible predictors representing different dimensions that may increase or decrease the need for ED visits. Information on all explanatory variables was obtained from the survey data.

#### Sociodemographic variables

The sociodemographic variables included age, gender, level of education (high / middle / low), and place of residence (home / round-the-clock care). Individuals who lived in private homes and in service housing with less than 24-hour assistance were categorized as living at home. Those who lived in service housing with 24-hour assistance, in a nursing home, and those whose place of stay at the time of the survey was a health center or hospital were categorized as living in round-the-clock care.

#### Care support

Care support comprised two variables: Living alone (yes / no) and receiving formal home care (yes / no).

#### Social contacts

Social contacts included two variables: frequency of meeting children (< 1 week ago / ≥ 1 week ago / do not have children), and frequency of speaking with a family member or friend over the phone (yesterday or today / < 1 week ago / ≥ 1 week ago).

#### Subjective health and wellbeing

The participants were asked about their self-rated health (SRH) (good / average / poor), life satisfaction (very satisfied / fairly satisfied / not satisfied), and feelings of tiredness and dizziness (yes often / yes sometimes / no). Proxy responses were excluded from these four subjective variables.

#### Functioning

Functioning was assessed with two questions on activities of daily living (ADL) (ability to get in and out of bed, dress and undress) and three questions on mobility (ability to walk 400 m, move around indoors, and climb stairs) based on Katz Index [23]. The response categories were, (1) yes without difficulty, (2) yes but with difficulty, (3) only with help, and (4) not at all. For each question, responses 1 and 2 were combined as independent and 3 and 4 as dependent. For the final analysis, ADL and mobility were further categorized as dependent in at least one activity or independent in all.

#### Sensory functions

Sensory functions were assessed with two questions: ability to read the newspaper (yes / partly / no) and ability to hear others talk (yes / partly / no).

#### Diseases

The 10 self-reported diagnosed diseases asked in the survey were heart disease (coronary heart disease, myocardial infarction, arrhythmias); hypertension; stroke; cancer; diabetes; dementia (Alzheimer’s disease, other dementia, or memory problems); Parkinson’s disease; depression (depression or depressive symptoms); hip fracture; and arthritis. These diseases were used to create the variable ‘number of chronic diseases’, which was categorized as 0–1, 2–3, or ≥ 4 conditions. The term multimorbidity is used to refer to the categories of 2–3 and ≥ 4 conditions” [3].

(For a detailed description of outcome and explanatory variables, see Supplementary file 1).

### Statistical analysis

Descriptive analyses are presented as means and standard deviations (SD) for continuous variables and as frequencies and percentages for categorical variables. Predictors of the first ED visit were examined as a time-to-event analysis (time to first ED visit) using Cox regression models and presented as hazard ratios (HR) with 95% confidence intervals (CI). Proportional hazards assumptions were tested and found to be violated for meeting children and SRH among those living at home, and for education, tiredness, and hearing among round-the-clock care residents. For these variables, a piecewise proportional hazards model was applied to estimate HRs with 95% CIs before and after the cut-point. A log–log survival curve was used to assess the cut-point and to divide the time axis into two intervals. For this analysis, ‘Hearing’ was recategorized as yes and no (combining no and partly) to increase the number of people in the category with hearing difficulty. Death was treated as a censoring event (coded as ‘0’). All individuals were followed until their first ED visit, death or until the end of follow-up (31st December 2017), whichever came first.

Predictors of the number of ED visits in one year of follow-up were analyzed with multinomial logistic regression models. The results are presented as relative risk ratios (RRR) and 95% CIs. Both analyses were stratified by place of residence (home / round-the-clock care), and each predictor variable was separately tested after age and gender adjustment in both analyses. A p-value of < 0.05 was considered statistically significant. All the analyses were performed using IBM SPSS version 26.

## Results

At baseline, three-fourths of the 1561 study participants were women. The mean age of the participants was 92.7 years, and more than half had a low level of education. Two-thirds lived at home and one-third in round-the-clock care.

The majority of the participants living at home were independent in ADL. Three-fifths were independent in mobility and around three-fourths could read and hear well. In addition, three-fourths lived alone, three-fifths did not receive formal home care, and one-fourth had at least four chronic diseases (For detailed information on individual diseases, see Supplementary file 2). Among participants living in round-the-clock care, two-fifths were independent in ADL and 17% in mobility, more than half and two-thirds, respectively, could read and hear well, and one-third had at least four chronic diseases. Slightly over one-third (38%) of those living at home and 13% of those living in round-the-clock care were alive at the end of the four-year follow-up (Table 1).

**Table 1:** Baseline and follow-up information on study participants by place of residence in the Vitality 90+ Study (2014)

Characteristic	Home	Round-the-clock care	Total
Baseline			
Age (*n* = 1561), mean ± SD*	92.3 ± 2.3	93.3 ± 3	92.7 ± 2.6
	*n* (%)	*n* (%)	*n* (%)
Gender (*n* = 1561)			
Male	260 (26.1)	109 (19.3)	369 (23.6)
Female	735 (73.9)	457 (80.7)	1192 (76.4)
Education (*n* = 1535)			
Low	489 (49.7)	309 (56.1)	798 (52.0)
Middle	322 (32.7)	168 (30.5)	490 (31.9)
High	173 (17.6)	74 (13.4)	247 (16.1)
Meeting children (*n* = 1539)			
No children	201 (20.5)	107 (19.1)	308 (20.0)
≥1 week	128 (13.1)	90 (16.1)	218 (14.2)
<1 week	651 (66.4)	362 (64.8)	1013 (65.8)
Talking to family or friends over phone (*n* = 1531)			
≥1 week	143 (14.5)	304 (55.9)	447 (29.2)
<1 week	174 (17.6)	77 (14.2)	251 (16.4)
Yesterday/today	670 (67.9)	163 (30.0)	833 (54.4)
Living alone (*n* = 994)			
Yes	757 (76.2)	-	757 (76.2)
No	237 (23.8)	-	237 (23.8)
Formal home help (*n* = 963)			
No	579 (60.1)	-	579 (60.1)
Yes	384 (39.9)	-	384 (39.9)
Self-rated health (SRH) (*n* = 1230)			
Poor	197 (21.2)	118 (39.5)	315 (25.6)
Average	433 (46.5)	131 (43.8)	564 (45.9)
Good	301 (32.3)	50 (16.7)	351 (28.5)
Life satisfaction (*n* = 1236)			
Not satisfied	106 (11.3)	61 (20.2)	167 (13.5)
Somewhat satisfied	566 (60.6)	180 (59.6)	746 (60.4)
Very satisfied	262 (28.1)	61 (20.2)	323 (26.1)
Tiredness (*n* = 1225)			
Yes, often	319 (34.4)	129 (43.1)	448 (36.6)
Yes, sometimes	556 (60.0)	157 (52.5)	713 (58.2)
No, never	51 (5.5)	13 (4.3)	64 (5.2)
Dizziness (*n* = 1236)			
Yes, often	253 (27.2)	102 (33.3)	355 (28.7)
Yes, sometimes	516 (55.5)	163 (53.3)	679 (54.9)
No, never	161 (17.3)	41 (13.4)	202 (16.3)
Activities of daily living (ADL) (*n* = 1550)			
Dependent	57 (5.8)	336 (60.1)	393 (25.4)
Independent	934 (94.2)	223 (39.9)	1157 (74.6)
Mobility (*n* = 1528)			
Dependent	402 (41.2)	462 (83.5)	864 (56.5)
Independent	573 (58.8)	91 (16.5)	664 (43.5)
Vision (able to read newspaper) (*n* = 1545)			
No	111 (11.2)	143 (25.7)	254 (16.4)
Partly	143 (14.5)	158 (28.4)	301 (19.5)
Yes	734 (74.3)	256 (46.0)	990 (64.1)
Hearing (able to hear others talk) (*n* = 1545)			
No	5 (0.5)	10 (1.8)	15 (1.0)
Partly	210 (21.3)	191 (34.2)	401 (26.0)
Yes	771 (78.2)	358 (64.0)	1129 (73.1)
Number of chronic diseases (*n* = 1546)			
≥ 4	248 (25.2)	194 (34.6)	442 (28.6)
2–3	509 (51.7)	297 (52.9)	806 (52.1)
0–1	228 (23.1)	70 (12.5)	298 (19.3)
Follow-up information			
Status at end of study (31st Dec 2017) (*n* = 1561)			
Dead	620 (62.3)	495 (87.5)	1115 (71.4)
Alive	375 (37.7)	71 (12.5)	446 (28.6)

*SD standard deviation

### ED visits

During the one year of follow-up, more than half of all, two-thirds of those living at home, and one-third of those living in round-the-clock care, had at least one ED visit. However, in the four years of follow-up, most of those living at home and more than half of those living in round-the-clock care had at least one ED visit. In the one year of follow-up, 16% of those living at home and 7% of round-the-clock care residents were frequent ED users, meaning that they had ≥ 4 ED visits in the one year of follow-up (Table 2).

**Table 2:** Emergency department visits among study participants in the Vitality 90+ Study (2014)

ED visits	Home (*n* = 995) *n* (%)	Round-the-clock care (*n* = 566) *n* (%)	Total (*n* = 1561) *n* (%)
ED visits during the study period (17 Jan 2014–31 Dec 2017)			
Yes	917 (92.2)	311 (54.9)	1228 (78.7)
No ED visit	78 (7.8)	255 (45.1)	333 (21.3)
ED visits in 1 year of follow-up (17 Jan 2014–16 Jan 2015)			
No visits	340 (34.2)	371 (65.5)	711 (45.5)
1–3	492 (49.4)	158 (27.9)	650 (41.6)
≥ 4	163 (16.4)	37 (6.5)	200 (12.8)

### Predictors of first ED visit

**Fig. 1:**
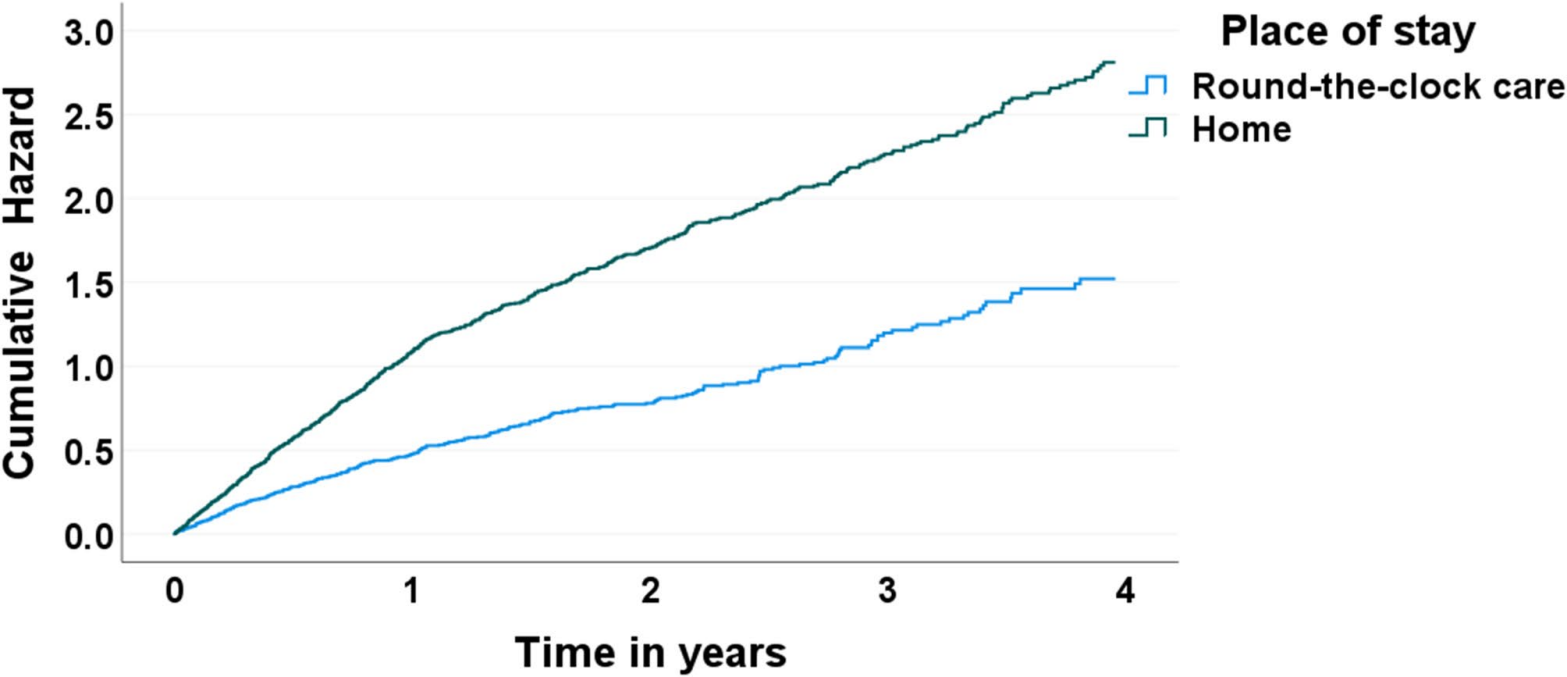
Cumulative hazard of first entry to emergency department by place of residence in the Vitality 90+ Study (2014–2017)

Cox regression models showed higher hazards of entering ED for those living at home compared to those living in round-the-clock care (Fig. 1). Figure 1 illustrates that those living at home had greater accumulated risk of having an ED visit than round-the-clock care residents over the four years. In both groups, the risk of entering ED was higher for men than women and among those with heart diseases, and lower among those who did not have children (Table 3). 

**Table 3:** Age and gender adjusted hazard of entering emergency department in the Vitality 90+ Study (2014–2017)

Explanatory variables	Home HR (95% CI)	Round-the-clock care HR (95% CI)
Sociodemographic factors		
Age	1.02 (0.99–1.05)	0.98 (0.94–1.02)
Gender, Male	**1.17 (1.01**–1.36)*	**1.39 (1.05**–1.82)*
Education^a^ (ref = High)		
Middle	0.98 (0.81–1.19)	
Before two years		0.92 (0.63–1.34)
After two years		1.13 (0.43–2.97)
Low	1.04 (0.86–1.24)	
Before two years		0.73 (0.51–1.04)
After two years		1.49 (0.62–3.54)
Care support		
Living alone, Yes	1.03 (0.87–1.21)	-
Formal home care, No	**1.33 (1.16**–1.52)***	-
Social contacts		
Meeting children^a^ (ref = < 1 week)		
≥ 1 week		**0.63 (0.44**–0.88)*
Before 10 months	0.91 (0.72–1.17)	
After 10 months	1.05 (0.75–1.47)	
Do not have any children		**0.62 (0.46**–0.84)*
Before 10 months	0.93 (0.75–1.14)	
After 10 months	0.66 (0.50–0.89)**	
Talking to family or friends over phone (ref = Yesterday/Today)		
< 1 week	0.88 (0.74–1.05)	0.81 (0.58–1.11)
≥ 1week	0.87 (0.72–1.05)	**0.44 (0.34**–0.56)***
Subjective health and wellbeing		
SRH^a^ (ref = Good)		
Average		0.87 (0.59–1.27)
Before two years	1.22 (1.03–1.44)*	
After two years	0.60 (0.40–0.90)*	
Poor		0.87 (0.58–1.28)
Before two years	1.74 (1.42–2.12)***	
After two years	0.92 (0.50–1.69)	
Life satisfaction (ref = Very satisfied)		
Somewhat satisfied	1.11 (0.95–1.29)	1.05 (0.74–1.48)
Not satisfied	**1.55 (1.23**–1.96)***	0.71 (0.45–1.13)
Tiredness^a^ (ref = No)		
Yes, sometimes	1.12 (0.83–1.51)	
Before one year		0.95 (0.41–2.18)
After one year		2.03 (0.49–8.39)
Yes, often	**1.59 (1.17**–2.16)*	
Before one year		0.97 (0.42–2.25)
After one year		1.22 (0.29–5.19)
Dizziness (ref = No)		
Yes, sometimes	0.96 (0.80–1.16)	1.14 (0.75–1.73)
Yes, often	**1.39 (1.13**–1.71)*	1.21 (0.78–1.89)
Functioning		
ADL, Dependent	**1.42 (1.08**–1.88)*	**0.46 (0.37**–0.58)***
Mobility, Dependent	**1.48 (1.29**–1.70)***	**0.54 (0.42**–0.71)***
Sensory functions		
Vision (able to read newspaper) (ref = Yes)		
Partly	**1.43 (1.19**–1.73)***	**0.69 (0.53**–0.90)*
No	1.18 (0.96–1.46)	**0.54 (0.39**–0.73)***
Hearing^a^ (able to hear others talk) (ref = Yes)		
No / partly	1.18 (1.01–1.39)*	
Before six months		0.82 (0.57–1.19)
After six months		0.48 (0.34–0.69)***
Diseases		
Hypertension, Yes	1.07 (0.94–1.23)	1.15 (0.92–1.44)
Heart Disease, Yes	**1.37 (1.20**–1.56)***	**1.63 (1.29**–2.06)***
Diabetes, Yes	1.12 (0.94–1.33)	1.07 (0.78–1.48)
Stroke, Yes	**1.40 (1.10**–1.78)*	0.90 (0.63–1.29)
Cancer, Yes	1.15 (0.97–1.36)	0.76 (0.54–1.07)
Dementia, Yes	1.04 (0.89–1.20)	**0.52 (0.42**–0.66)***
Parkinson’s, Yes	**2.13 (1.17**–3.89)*	0.54 (0.20–1.44)
Hip fracture, Yes	**1.29 (1.08**–1.55)*	1.18 (0.91–1.52)
Depression, Yes	1.20 (0.99–1.45)	0.88 (0.67–1.15)
Arthritis, Yes	1.05 (0.92–1.20)	**1.26 (1.00**–1.58)*
Number of chronic diseases (ref = 0–1)		
2–3	**1.26 (1.07**–1.48)**	1.06 (0.74–1.52)
≥ 4	**1.51 (1.25**–1.82)***	1.05 (0.72–1.54)

*Abbreviations: HR *hazard ratio,* CI *confidence interval, from Cox regression models

*p < 0.05, **p < 0.01, ***p < 0.001; bold text indicates statistical significance

^a^For variables where the proportional hazard assumption was not met, results are presented with two-time intervals (detailed description provided in Statistical analysis section)

Among those living at home, several factors increased the risk of entering ED: not receiving formal home care, poor SRH, being unsatisfied with life, frequent tiredness and dizziness, dependence in ADL and mobility, only partial ability to read, and impaired hearing. In addition, stroke, hip fracture, Parkinson’s disease, and multimorbidity were associated with an increased risk of entering ED. Among those living in round-the-clock care, on the other hand, we found several factors that decreased the risk of entering ED: fewer social contacts, having dementia, dependence in ADL and mobility, and impaired sensory functions. The presence of arthritis was associated with an increased risk of entering ED among those living in round-the-clock care (Table 3).

### Predictors of 1−3 and ≥ 4 ED visits in one year of follow-up (reference 0 ED visits)

With few exceptions, the predictors of having 1−3 or ≥ 4 ED visits in one year of follow-up differed between those living at home and those living in round-the-clock care. Having heart disease was associated with 1−3 and ≥ 4 ED visits independent of place of residence, and less frequent social contact was associated with a reduced likelihood of having ED visits, especially among those in round-the-clock-care (Table 4).

**Table 4:** Multinomial logistic regression models for number of emergency visits during one-year follow-up (2014–2015)

Explanatory variables	Home		Round-the-clock care	
	1–3 ED visits RRR (95%CI)	≥ 4 ED visits RRR (95%CI)	1–3 ED visits RRR (95%CI)	≥ 4 ED visits RRR (95%CI)
Sociodemographic factors				
Age	1.04 (0.97–1.10)	0.98 (0.90–1.06)	0.94 (0.89–1.01)	0.90 (0.79–1.03)
Gender, male	1.31 (0.95–1.80)	1.38 (0.91–2.11)	**1.61 (1.02**–2.54)*	1.88 (0.87–4.09)
Education (High)				
Middle	0.90 (0.60–1.37)	1.33 (0.75–2.36)	0.89 (0.49–1.62)	1.19 (0.40–3.57)
Low	1.01 (0.69–1.49)	1.26 (0.72–2.18)	0.74 (0.42–1.30)	0.90 (0.31–2.60)
Care support				
Living alone, Yes	1.06 (0.74–1.51)	1.20 (0.74–1.95)	-	–
Formal home care, No	1.39 (1.02–1.88)*	**1.89 (1.27**–2.81)*	-	–
Social contacts				
Meeting children (≤ 1 week)				
≥1 week	1.35 (0.88–2.06)	**0.40 (0.19**–0.87)*	**0.45 (0.25**–0.81)*	0.45 (0.15–1.35)
No children	0.77 (0.54–1.10)	0.84 (0.53–1.33)	**0.46 (0.27**–0.79)*	0.51 (0.19–1.36)
Talking to family or friends over phone (Today/ Yesterday)				
<1 week	1.06 (0.73–1.54)	0.89 (0.53–1.48)	0.85 (0.47–1.53)	0.60 (0.25–1.48)
≥1 week	0.87 (0.59–1.30)	0.56 (0.31–1.02)	**0.38 (0.24**–0.58)***	**0.06 (0.02**–0.17)***
Subjective health and wellbeing				
SRH (Good)				
Average	1.33 (0.96–1.84)	**1.73 (1.09**–2.74)*	0.87 (0.43–1.76)	0.97 (0.28–3.35)
Poor	**2.02 (1.33**–3.08)**	**3.02 (1.73**–5.26)***	0.73 (0.35–1.50)	1.25 (0.37–4.21)
Life satisfaction (Very satisfied)				
Somewhat satisfied	**1.43 (1.04**–1.98)*	1.01 (0.66–1.55)	1.14 (0.61–2.16)	1.46 (0.50–4.21)
Not satisfied	**2.15 (1.25**–3.70)*	**2.21 (1.14**–4.27)*	0.70 (0.32–1.56)	0.69 (0.17–2.81)
Tiredness (No)				
Yes sometimes	1.38 (0.76–2.50)	**3.51 (1.03**–11.95)*	0.92 (0.28–3.08)	1.01 (0.11–9.02)
Yes often	2.02 (1.08–3.78)*	**6.13 (1.77**–21.25)*	0.76 (0.22–2.57)	1.43 (0.16–12.70)
Dizziness (No)				
Yes sometimes	1.01 (0.69–1.49)	1.23 (0.70–2.14)	0.79 (0.37–1.68)	0.42 (0.14–1.27)
Yes often	**1.76 (1.13**–2.76)*	**2.47 (1.33**–4.59)*	0.84 (0.38–1.87)	0.66 (0.21–2.05)
Functioning				
ADL, Dependent	1.43 (0.78–2.66)	1.06 (0.44–2.54)	**0.37 (0.25**–0.55)***	**0.20 (0.10**–0.43)***
Mobility, Dependent	**1.45 (1.07**–1.95)*	**2.15 (1.44**–3.19)***	**0.45 (0.28**–0.75)*	**0.29 (0.13**–0.63)*
Sensory functions				
Vision (able to read newspaper) (Yes)				
Partly	**1.55 (1.02**–2.35)*	1.65 (0.95–2.89)	0.65 (0.42–1.02)	0.73 (0.33–1.65)
No	1.04 (0.65–1.67)	**2.02 (1.17**–3.50)*	**0.45 (0.27**–0.74)*	0.52 (0.21–1.29)
Hearing (able to hear others talk) (Yes)				
Partly	1.23 (0.87–1.75)	1.51 (0.96–2.38)	0.72 (0.48–1.09)	0.58 (0.26–1.28)
No	1.94 (0.20–18.94)	2.36 (0.14–38.60)	0.55 (0.11–2.65)	-
Diseases				
Hypertension, Yes	1.08 (0.81–1.45)	**1.66 (1.09**–2.51)*	1.18 (0.80–1.72)	1.66 (0.81–3.38)
Heart Disease, Yes	**1.53 (1.15**–2.02)*	**2.00 (1.36**–2.94)***	**1.49 (1.02**–2.19)*	**3.38 (1.50**–7.61)*
Diabetes, Yes	0.89 (0.61–1.30)	1.01 (0.61–1.66)	1.21 (0.70–2.07)	1.80 (0.77–4.21)
Stroke, Yes	1.59 (0.88–2.86)	**2.77 (1.42**–5.42)*	0.98 (0.54–1.78)	0.91 (0.30–2.71)
Cancer, Yes	1.40 (0.96–2.05)	1.20 (0.72–2.01)	1.86 (0.85–4.08)	0.45 (0.13–1.52)
Dementia, Yes	1.07 (0.78–1.47)	0.78 (0.50–1.21)	**0.59 (0.39**–0.88)*	**0.12 (0.05**–0.25)***
Parkinson’s, Yes	2.60 (0.54–12.37)	1.84 (0.26–13.28)	0.75 (0.20–2.82)	-
Hip fracture, Yes	1.41 (0.93–2.16)	1.75 (1.03–2.99)*	1.36 (0.88–2.10)	0.70 (0.28–1.75)
Depression, Yes	1.28 (0.82–1.99)	**1.81 (1.06**–3.11)*	1.14 (0.74–1.75)	0.99 (0.45–2.20)
Arthritis, Yes	1.11 (0.84–1.47)	1.11 (0.76–1.62)	1.26 (0.86–1.85)	1.66 (0.83–3.29)
Number of chronic diseases (0–1)				
2–3	**1.54 (1.09**–2.16)*	**1.64 (1.01**–2.68)*	1.40 (0.74–2.64)	1.01 (0.36–2.85)
≥4	**1.82 (1.21**–2.73)**	**2.36 (1.36**–4.09)**	1.63 (0.84–3.16)	0.93 (0.31–2.81)

Reference category = individuals who do not have ED visits

*Abbreviations: RRR* relative risk ratio, *CI* confidence interval

*p < 0.05, **p < 0.01, ***p < 0.001; bold text indicates statistical significance

Among those living at home, most indicators of poor subjective health and wellbeing, lack of formal home care, and multimorbidity increased the probability of having 1–3 and ≥ 4 ED visits. In addition, individuals with a history of hypertension, stroke, and depression were more likely to have ≥ 4 ED visits instead of not having ED visits. Dependence in mobility and impaired vision increased the probability of having a higher number of ED visits among those living at home but decreased the probability among those living in round-the-clock care. Indeed, several factors were associated with a decreased probability of having 1–3 and ≥ 4 ED visits among round-the-clock care residents, including dependence in ADL and having dementia. Furthermore, men in round-the-clock-care were more likely than women to have 1–3 ED visits instead of not having ED visits (Table 4).

## Discussion

In this population-based study, we examined the frequency and predictors of ED visits among the oldest old individuals living at home and in round-the-clock care in Tampere, Finland. The results show that during a one-year follow-up more than half, and during a four-year follow-up eight out of ten study participants, had at least one ED visit. One out of eight persons were frequent ED users, having at least four ED visits a year. Those living at home used ED more frequently, and they were found to have several health, functioning, and social relations characteristics that increased the risk of entering ED. Among those living in round-the-clock care, we mainly identified factors that reduced the frequency of visits to ED.

### ED visits and frequent ED use

Even though previous studies have not compared the frequency of ED visits between the oldest old living at home and those in round-the-clock care, some findings have been reported for both these groups separately from different contexts and among the broader 65 + group. In this study, 66% of home dwellers, 34% of round-the-clock care residents, and 55% in total had at least one ED visit in one year of follow-up. A Swedish community-based study found that 36% of the 80 + population had at least one ED visit in a year [24]. Two studies from the United States have reported corresponding proportions of 47% [25] and 62% [26] for people aged over 65 and living in a nursing home. Since health and care systems differ between countries, country comparisons are challenging. However, in this study, the frequency of ED visits was higher in total, and among those living in the community, but clearly lower among round-the-clock care residents than reported in prior studies. Most of the round-the-clock care residents in this age group have dementia and are approaching the end of life, and they require continuous supportive and palliative care. That care is provided by trained staff, which reduces the need for ED.

Our results showed a higher proportion of frequent ED users than previous reports: 12.8% in total, 16.4% among home dwellers, and 6.5% among round-the-clock care residents. A Finnish study found that of ED users 80 years and over, 8.2% were frequent ED users [4], while studies among people aged 65 + reported figures of 5–6.6% for Australian ED attendees [17] and community-dwelling Canadians [16]. The higher frequency of ED visits seen here compared to previous studies with younger participants is unsurprising given that health deteriorates and care needs increase with age.

### Predictors of ED visits

Among the home-dwellers in our study, three-fourths lived alone and 40% received formal home care. In contrast to earlier findings [15, 24], living alone did not increase the risk of using ED, but not receiving formal home care did. The care policy, which aims to promote living at home for as long as possible, has led to a declining coverage of round-the-clock care [27], but the availability of home care services has not increased accordingly [28]. Increased ED use among those not receiving formal home care could be due to problems with access to or the availability of home care services [29], leading to unmet care needs. Given the prolonged waiting times to primary health care in Finland today, ED might be the most accessible entry point into health care services, even with non-acute care needs [30]. Especially among round-the-clock care residents, less frequent social contact was associated with a lower frequency of ED visits. In the case of the oldest old living in round-the-clock care with dementia, visiting family members are likely to detect any deterioration in their health and to seek urgent intensive care, which may well increase the frequency of ED visits. However, more research is needed to throw light on the role of social contacts, especially family members, in the use of emergency care services among older people.

Among the home-dwellers in our study, indicators of subjective health and wellbeing such as poor SRH, poor life satisfaction, and frequent tiredness and dizziness were associated with a higher risk of entering ED and being a frequent ED user. Poor SRH has been identified as a predictor of ED visits in many other studies as well [31–34]. Weakness, dizziness, and fatigue have also been associated with increased ED visits [35, 36]. The strong association of subjective health indicators with ED visits may reflect underlying diseases, disability, and lack of social contacts and support [35, 37, 38] and adversely affect health, recovery, and health services use [31, 38, 39].

In line with earlier studies [14, 32, 40], we found that heart diseases increased the risk of entering ED and being a frequent ED user among both home dwellers and round-the-clock care residents. Similarly to previous reports, our study showed that other chronic conditions such as depressive symptoms [31, 34, 40], stroke and hip fracture [40] and multimorbidity [41] seem to increase these risks only among those living at home. In keeping with the results from a systematic review [41], we found that among home dwellers, impaired functioning and sensory functions were associated with higher ED use. In contrast to home dwelling individuals, impairments in functioning and sensory functions decreased the frequency of visits to ED among round-the-clock residents. The higher the level of functional impairment, the closer the person is to death, which raises the threshold for ED admissions.

Some studies [41] suggest that cognitive impairment increases the frequency of ED visits, but we found that having dementia or memory problems decreased ED visits for those in round-the-clock care and had no effect for home dwellers. Morris et al. [32] and Dufour et al. [16] reported similar results among home dwellers. Home dwellers are likely to have mild to moderate dementia and sufficient health and social support because they can live independently. However, they might also struggle to seek help due to their condition. Prior research indicates that nursing home residents with mild cognitive impairment have higher ED visit risks, while those with severe impairment have reduced risks [26]. As people live at home for as long as possible, very old people move to round-the-clock care with complex chronic conditions and severe dementia. It is likely that only those who require intensive care and who cannot be taken care of at a round-the-clock care facility will be transferred to health care [16, 42].

### Strengths and limitations

The main strengths of this study are its large population-based cohort, high response rate (80%), and data linkages with exhaustive national registers, which ensured complete follow-up. Although restricted to a single geographical area, the study population includes the oldest old living at home and in round-the-clock care in both urban and rural areas. By using proxy respondents, we were able to reach the whole spectrum of oldest old individuals from those living independently and being cognitively intact to individuals with cognitive impairments and requiring 24-hour care. Proxy responses, however, were not included in the analyses of subjective wellbeing, which means that the results represent the healthier end of the population. One limitation in our study, as in most population-based studies, is its reliance on self-reported data. However, based on our recent study that compared survey information with national register data on chronic conditions, the level of agreement is acceptable among this oldest old population [43]. Another limitation is the restricted number of survey questions in our study, a conscious choice we made to guarantee a high response rate and to keep the questionnaire similar between the survey years. Furthermore, we do not know whether some of the individuals who initially lived at home moved to round-the-clock care during the follow-up before their first ED visit.

## Conclusion

This study showed that the frequency and predictors of ED visits greatly differ between the oldest old individuals living at home and in round-the-clock care. We identified several factors that increased the risk of entering ED among home dwellers and some that decreased the risk among round-the-clock care residents. Due to the declining coverage of round-the-clock care, more people with mild to moderate dementia and disability are now living in their own homes. Since most ED visits occurred among those living at home and having poor health but not receiving formal home care, improving the continuity of care and the coverage of home care services could help to curb the increase in ED visits among the fast-growing oldest old population.

## Supplementary Information

Supplementary Material 1.

Supplementary Material 2.

## Data Availability

The Vitality 90+ Study survey data are described in an open access repository called the Finnish Social Sciences Data Archive, Vitality 90+| Aila Data Service (tuni.fi) (https://services.fsd.tuni.fi/catalogue/series/64?lang=en). Access to the survey data are available from the research group on reasonable request. The national register data on emergency department visits were retrieved from the Finnish Institute for Health and Welfare (Dnro THL/6209/14.02.00/2023). Due to GDPR regulations and the sensitive nature of the data, there are restrictions on its availability.

## Abbreviations

ADL: Activities of daily living
CI: Confidence interval
ED: Emergency department
HR: Hazard ratio
RRR: Relative risk ratio
SD: Standard deviation
SRH: Self-rated health
THL: Finnish Institute for Health and Welfare

## References

[1] World Population Prospects. 2024. United Nations Population Division. https://population.un.org/dataportal/data/indicators/70,71/locations/900/start/1990/end/2020/table/pivotbyage?df=b9e2999d-b2a0-4457-90e9-fddd3cf74814. Accessed 30 Oct 2024.

[2] Statistics Finland. population structure. https://pxdata.stat.fi/PxWeb/pxweb/en/StatFin/StatFin__vaerak/statfin_vaerak_pxt_11rd.px/. Accessed 17 Dec 2024.

[3] Skou ST, Mair FS, Fortin M, Guthrie B, Nunes BP, Miranda JJ, Boyd CM, Pati S, Mtenga S, Smith SM. Multimorbidity. Nat Rev Dis Primers. 2022;8(1):48. 10.1038/s41572-022-00376-4. PMID: 35835758; PMCID: PMC7613517. 35835758 10.1038/s41572-022-00376-4 PMC7613517

[4] Ukkonen M, Jämsen E, Zeitlin R, Pauniaho S. Emergency department visits in older patients: a population-based survey. BMC Emerg Med. 2019;19:20. 30813898 10.1186/s12873-019-0236-3 PMC6391758

[5] Abtan R, Rotondi NK, Macpherson A, Rotondi MA. The effect of informal caregiver support on utilization of acute health services among home care clients: a prospective observational study. BMC Health Serv Res. 2018;8:73. 10.1186/s12913-018-2880-9. 10.1186/s12913-018-2880-9 PMC5793410 29386027

[6] Namara RMc, Irepel D, Ferrgusson R, Glaser K. Determinants in older adults who frequently attend the emergency department. Age Ageing. 2018;47:v13–60. 10.1093/ageing/afy140.38.

[7] Covino M, Petruzziello C, Onder G, Migneco A, Simeoni B, Franceschi F, Ojetti V. A 12-year retrospective analysis of differences between elderly and oldest old patients referred to the emergency department of a tertiary hospital. Maturitas. 2019;120:7–11. 30583768 10.1016/j.maturitas.2018.11.011

[8] Gruneir A, Silver MJ, Rochon PA. Review: Emergency department use by older adults: A literature review on trends, appropriateness, and consequences of unmet health care needs. Med Care Res Rev. 2011;68(2):131–55. 10.1177/1077558710379422. 20829235 10.1177/1077558710379422

[9] Crimmins EM, Shim H, Zhang YS, Kim JK. Differences between men and women in mortality and the health dimensions of the morbidity process. Clin Chem. 2019;65(1):135–45. 30478135 10.1373/clinchem.2018.288332 PMC6345642

[10] Carmel S. Health and Well-Being in late life: gender differences worldwide. Front Med. 2019;6:218. 10.3389/fmed.2019.00218. 10.3389/fmed.2019.00218 PMC6795677 31649931

[11] Haapamäki E, Huhtala H, Löfgren T, Mylläri E, Seinelä L, Valvanne J. Elderly people as emergency room users: emergency room visits of Tampere residents aged 70 and over in 2011–2012. (Publications of the Elderly Population Services: Use, Costs, Impact and Financing Project; No. 3). Association of Finnish Local and Regional Authorities 2014. https://www.google.com/url?sa=t&rct=j&q=&esrc=s&source=web&cd=&cad=rja&uact=8&ved=2ahUKEwitj5yAibmNAxWFRvEDHeCvG2YQFnoECBUQAQ&url=https%3A%2F%2Fjulkaisut.kuntaliitto.fi%2F1650&usg=AOvVaw1CyQcKhBYDCzMOIKQrJx4A&opi=89978449. Accessed 22 May 2025.

[12] Enroth L, Fors S. Trends in the social class inequalities in disability and self-rated health: repeated cross-sectional surveys from Finland and Sweden 2001–2018. Int J Public Health. 2021;66:645513. 10.3389/ijph.2021.645513. 34744593 10.3389/ijph.2021.645513 PMC8565263

[13] Doheny M, Agerholm J, Orsini N, Schon P, Burstrom B. Socio-demographic differences in the frequent use of emergency department care by older persons: a population-based study in Stockholm County. BMC Health Serv Res. 2019;19:202. 30922354 10.1186/s12913-019-4029-x PMC6440084

[14] Dufour I, Chouinard M-C, Dubuc N, Beaudin J, Lafontaine S, Hudon C. Factors associated with frequent use of emergency-department services in a geriatric population: a systematic review. BMC Geriatr. 2019;19:185. 31277582 10.1186/s12877-019-1197-9 PMC6610907

[15] Dreyer K, Steventon A, Fisher R, Deeny SR. The association between living alone and health care utilisation in older adults: a retrospective cohort study of electronic health records from a London general practice. BMC Geriatr. 2018;18:269. 30514225 10.1186/s12877-018-0939-4 PMC6280341

[16] Dufour I, Chiu Y, Courteau J, Chouinard M-C, Dubuc N, Hudon C. Frequent emergency department use by older adults with ambulatory care sensitive conditions: A population-based cohort study. Geriatr Gerontol Int. 2020;20:317–23. 10.1111/ggi.13875. 32017348 10.1111/ggi.13875 PMC7187263

[17] Street M, Berry D, Considine J. Frequent use of emergency departments by older people: a comparative cohort study of characteristics and outcomes. Int J Qual Health Care. 2018;30(8):624–9. 29659863 10.1093/intqhc/mzy062

[18] Vilpert S, Ruedin HJ, Trueb L, Monod-Zorzi S, Yersin B, Bula C. Emergency department use by oldest-old patients from 2005 to 2010 in a Swiss university hospital. BMC Health Serv Res. 2013;13:344. 24011089 10.1186/1472-6963-13-344 PMC3846121

[19] Olsen C, Pedersen I, Bergland A, Enders-Slegers M, Jøranson N, Calogiuri G, Ihlebaek C. Differences in quality of life in home-dwelling persons and nursing home residents with dementia– a cross sectional study. BMC Geriatr. 2016;6:137. 10.1186/s12877-016-0312-4 PMC4939817 27400744

[20] Trivedi S, Roberts C, Karreman E, Lyster K. Characterizing the long-term care and community-dwelling elderly patient’s use of the emergency department. Cureus. 2018;10,11:e3642. 10.7759/cureus.3642. 30705794 10.7759/cureus.3642 PMC6349572

[21] Enroth L, Halonen P, Tiainen K. Cohort profile: the vitality 90+ Study– a cohort study on health and living conditions of the oldest old in Tampere, Finland. BMJ Open. 2023;13:e068509. 10.1136/bmjopen-2022-068509. 36750290 10.1136/bmjopen-2022-068509 PMC9906174

[22] HealthVillage.Fi. Information on emergency and paramedical care. Glossary for emergency medicine. https://www.terveyskyla.fi/en/emergencyhub/information-about-emergency-and-paramedical-care/glossary-for-emergency-medicine. Accessed 20 Dec 2024.

[23] Katz S, Ford AB, Moskowitz RW, Jackson BA, Jaffe MW. Studies of illness in the aged: the index of ADL: A standardized measure of biological and psychological function. JAMA. 1963;185(12):914–9. 10.1001/jama.1963.03060120024016. 14044222 10.1001/jama.1963.03060120024016

[24] Naseer M, McKee KJ, Ehrenberg A, Schön P, Dahlberg L. Individual and contextual predictors of emergency department visits among community-living older adults: a register-based prospective cohort study. BMJ Open. 2022;12:e055484. 10.1136/bmjopen-2021-055484. 35140159 10.1136/bmjopen-2021-055484 PMC8830250

[25] LaMantia MA, Lane KA, TuW, Carnahan JL, Messina F, Unroe KT. Patterns of emergency department use among long-stay nursing home residents with differing levels of dementia severity. J Am Med Dir Assoc. 2016;17(6):541–6. 27052563 10.1016/j.jamda.2016.02.011 PMC4884504

[26] Stephens CE, Newcomer R, Blegen M, Miller B, Harrington C. Emergency department use by nursing home residents: effect of severity of cognitive impairment. Gerontologist. 2011;52(3):383–93. 22056961 10.1093/geront/gnr109

[27] Ministry of Social Affairs and Health. Quality recommendation to guarantee a good quality of life and improved services for older persons 2020–2023. The Aim is an Age-friendly Finland. 2020;37:1–76. http://urn.fi/URN:ISBN:978-952-00-8427-1. Accessed 18 Dec 2024.

[28] Saukkonen S, Marttila T. Kotihoito 2022. Kotihoidon Käynti- ja Asiakasmäärä Väheni Vuonna 2022 [Home Care 2022. The Amount of Home Care Visits and Clients Reduced in 2022], Statistic Report 28/2023, Helsinki: National Institute of Health and Welfare. 2023. https://urn.fi/URN:NBN:fi-fe2023052648789.

[29] Ismail S, Thorlby R, Holder H. Focus on: social care for older people. London: The Health Foundation, Nuffield Trust.; 2014.

[30] Tervola J, Aaltonen K, Tallgren F. Can people afford to pay for health care? New evidence on financial protection in Finland. Copenhagen: WHO Regional Office for Europe; 2021.

[31] Chandra A, Crane SJ, Tung EE, Hanson GJ, North F, Cha SS, Takahashi PY. Patient-Reported geriatric symptoms as risk factors for hospitalization and emergency department visits. Aging Disease. 2015;6(13):188–95. 26029477 10.14336/AD.2014.0706 PMC4441401

[32] Morris JN, Howard EP, Steel K, Schreiber R, Fries BE, Lipsitz LA, Goldman B. Predicting risk of hospital and emergency department use for home care elderly persons through a secondary analysis of cross-national data. BMC Health Serv Res. 2014;14:519. 25391559 10.1186/s12913-014-0519-z PMC4236798

[33] Neufeld E, Viau KA, Hirdes JP, Warry W. Predictors of frequent emergency department visits among rural older adults in Ontario using the resident assessment Instrument-Home care. Aust J Rural Health. 2016;24:115–22. 26123034 10.1111/ajr.12213

[34] Mowbray FI, Aryal K, Mercier E, Heckman G, Costa AP. Older emergency department patients: does baseline care status matter?? Can Geriatr J. 2020;23(4):289–96. 33282049 10.5770/cgj.23.421 PMC7704072

[35] Bhalla MC, Wilber ST, Stiffler KA, Ondrejka JE, Gerson LW. Weakness and fatigue in older ED patients in the united States. Am J Emerg Med. 2014;32(11):1395–8. 25205614 10.1016/j.ajem.2014.08.027

[36] Simon NR, Jauslin AS, Bingisser R, Nickel CH. Emergency presentations of older patients living with frailty: presenting symptoms compared with non-frail patients. Am J Emerg Med. 2022;59:111–7. 35834872 10.1016/j.ajem.2022.06.046

[37] Calderón-Larrañaga A, Vetrano DL, Welmer A, Grande G, Fratiglioni L, Dekhtyar S. Psychological correlates of Multimorbidity and disability accumulation in older adults. Age Ageing. 2019;48:789–96. 31579908 10.1093/ageing/afz117 PMC6814086

[38] Schneider A, Riedlinger D, Pigorsch M, Holzinger F, Deutschbein J, Keil T, Möckel M, Schenk L. Self-reported health and life satisfaction in older emergency department patients: socio-demographic, disease-related and care-specific associated factors. BMC Public Health. 2021;21:1440. 10.1186/s12889-021-11439-8. 34289829 10.1186/s12889-021-11439-8 PMC8296655

[39] Tamayo-Fonseca N, Nolasco A, Quesada JA, Pereyra-Zamora P, Melchor I, Moncho J, Calabuig J, Barona C. Self-rated health and hospital services use in the Spanish National health system: a longitudinal study. BMC Health Serv Res. 2015;15,492. 10.1186/s12913-015-1158-8. 10.1186/s12913-015-1158-8 PMC4634188 26537822

[40] Kong D, Wong YI, Wang J, Sun BC, Dong X. Correlates of emergency department service utilization among U.S. Chinese older adults. J Immigr Minor Health. 2019;21:938–45. 30302622 10.1007/s10903-018-0828-0 PMC6639148

[41] Aminzadeh F, Dalziel WB. Older adults in the emergency department: A systematic review of patterns of use, adverse outcomes, and effectiveness of interventions. Ann Emerg Med. 2002;39:238–47. 11867975 10.1067/mem.2002.121523

[42] Hunt LJ, Coombs LA, Stephens CE. Emergency department use by Community-Dwelling individuals with dementia in the united States: an integrative review. J Gerontol Nurs. 2018;44(3):23–30. 29355877 10.3928/00989134-20171206-01 PMC5982587

[43] Halonen P, Jämsen E, Enroth L, Jylhä M. Agreement between self-reported information and health register data on chronic diseases in the oldest old. Clin Epidemiol. 2023;785–94. 10.2147/CLEP.S410971. 10.2147/CLEP.S410971 PMC10312216 37396023

