# Supplementary material for: Frequency and predictors of emergency department visits among the oldest old in Finland: the Vitality 90+ Study
Source: BMC Health Serv Res. 2025 Jun 5;25:807. doi: 10.1186/s12913-025-12923-2 (PMC12139172; doi:10.1186/s12913-025-12923-2)
Supplement: Supplementary file 1 — Supplementary Material 1. [file 12913_2025_12923_MOESM1_ESM.docx]

**Supplementary file 1**

Description of variables in the Vitality 90+ Study linked with national register data on emergency department visits

| **Variable name** | **Description of variable** |
| --- | --- |
| **Outcome variable from register data** | |
| Emergency Department  (ED) visits | ED provides round-the-clock care for patients whose health could be compromised by delay in health care. ED visits include both primary and specialized care, both facilities being located at the same collaborative ED unit of the university hospital in Tampere.  Data obtained from the care register for health care and the register for primary health care visits. In these registers, ED visits include visits to the emergency department, visits recorded as urgent need for care, immediate need for care, and urgent care in another unit. The codes for each of these were combined to get the total number of ED visits for every individual.  Time to first ED visit (in 3 years 11 months): 0) No ED visit, 1) ED visit present.  In 1-year follow-up: 1) No ED visit, 2) 1–3 ED visits, 3) four or more ED visits (frequent ED user). |
|  | |
| **Independent variables from the Vitality 90+ survey data** | |
| **I. Sociodemographic variables** | |
| Age | Age in full years  treated as a continuous variable |
| Gender | 1) Male and 2) Female |
| Education | Highest attained level of education  1 primary school, 2 lower secondary school, 3 vocational education, 4 folk high school, 5 high school/upper secondary, 6 college level, 7 university education  → 1) low (primary school), 2) middle (lower secondary school, vocational education, folk high school), 3) high (upper secondary, college level, university education) |
| Place of residence | Where do you live/ What is your place of residence when you answer the questionnaire?  1 Home, 2 service housing < 24h assistance, 3 service housing with 24h assistance, 4 nursing home, 5 health center/ hospital  → 1) round-the-clock care (service housing with 24h assistance, nursing home, health center/ hospital), 2) Home (home, service housing < 24h assistance) |
|  | |
| **II. Care support** | |
| Living alone | Who do you live with?  1 alone, 2 spouse, 3 children, 4 grandchildren, 5 someone else  → 1) live alone, 2) live with someone |
| Availability of formal home care | Does a home care worker visit you at least once a week?  1) no, 2) yes |
|  | |
| **III. Social contacts** | |
| Meeting children | When was the last time you met with any of your children?  1 I do not have children, 2 today or yesterday, 3 a couple of days but less than a week ago, 4 a week or two ago, 5 several weeks ago, 6 several months ago, 7 several years ago  → 1) no children, 2) ≥1 week ago, 3) < 1 week ago. |
| Talking to family or friends over phone | When was the last time you spoke on the phone with a family member, relative or friend?  1 today or yesterday, 2 a couple of days but less than a week ago, 3 a week or two ago, 4 several weeks ago, 5 several months ago, 6 several years ago  → 1) ≥1week ago, 2) < 1 week ago, 3) today / yesterday |
|  | |
| **IV. Subjective health and wellbeing** | |
| Self-rated health (SRH) | How would you evaluate your current health status?  1 Very good, 2 fairly good, 3 average, 4 fairly poor, 5 poor  → 1) poor (fairly poor, poor), 2) average, 3) good (very good, fairly good) |
| Life satisfaction | How satisfied are you with your current life?  1 very satisfied, 2 somewhat satisfied, 3 not very satisfied, 4 not at all satisfied  → 1) not satisfied (not very satisfied, not at all satisfied), 2) somewhat satisfied, 3) very satisfied |
| Tiredness | Do you feel tired?  1) Yes often, 2) yes sometimes, 3) no never |
| Dizziness | Are you dizzy or do you feel you have poor balance?  1) Yes often, 2) yes sometimes, 3) no never |
|  | |
| **V. Functioning** | |
| Activities of daily living (ADL) | Are you able to dress and undress?  Are you able to get in and out of bed?  1 yes without difficulty, 2 yes but with difficulty, 3 only with help, 4 not at all.  → 1 Dependent (only with help and not at all), 2 Independent (yes without difficulty, yes with difficulty)  ADL: 1) dependent in at least one activity, 2) independent in both |
| Mobility | Are you able to move indoors?  Are you able to walk 400 meters?  Are you able to climb stairs?  1 yes without difficulty, 2 yes but with difficulty, 3 only with help, 4 not at all.  → 1 Dependent (only with help and not at all), 2 Independent (yes without difficulty, yes with difficulty)  Mobility: 1) dependent in at least one activity, 2) independent in all three |
|  | |
| **VI. Sensory Functions** | |
| Vision | Can you read the newspaper (with glasses if you use them)?  1) no, 2) yes partly, 3) yes |
| Hearing | Can you hear other persons talk if you are alone with that person (using a hearing aid if you use it)?  1) no, 2) yes partly, 3) yes |
|  | |
| **VII. Diseases** | |
| Chronic Disease | Have you been diagnosed by your doctor as having any of the following diseases? 1 Yes, 2 No  heart disease (coronary heart disease, myocardial infarction, or arrhythmias);  hypertension;  stroke;  cancer;  diabetes;  dementia (Alzheimer’s disease, other dementia, or memory problems);  Parkinson’s disease;  depression (depression or depressive symptoms);  hip fracture;  or arthritis.  Number of chronic diseases: 1) 0–1, 2) 2–3, 3) ≥4 |
