# Supplementary material for: Frequency and predictors of emergency department visits among the oldest old in Finland: the Vitality 90+ Study
Source: BMC Health Serv Res. 2025 Jun 5;25:807. doi: 10.1186/s12913-025-12923-2 (PMC12139172; doi:10.1186/s12913-025-12923-2)
Supplement: Supplementary file 2 — Supplementary Material 2. [file 12913_2025_12923_MOESM2_ESM.docx]

Supplementary file 2

Baseline disease status of study participants by place of residence in the Vitality 90+ Study (2014)

| **Disease** | **Home**  **985 (%)** | **Round-the-clock care**  **561 (%)** | **Total**  **1546 (%)** |
| --- | --- | --- | --- |
| Hypertension | 629 (63.9) | 310 (55.3) | 939 (60.7) |
| Heart Disease | 518 (52.6) | 313 (55.8) | 831 (53.8) |
| Diabetes | 160 (16.2) | 77 (13.7) | 237 (15.3) |
| Stroke | 77 (7.8) | 63 (11.2) | 140 (9.1) |
| Cancer | 173 (17.6) | 82 (14.6) | 255 (16.5) |
| Dementia | 264 (26.8) | 392 (69.9) | 656 (42.4) |
| Parkinson’s | 12 (1.2) | 12 (2.1) | 24 (1.6) |
| Hip fracture | 138 (14.0) | 129 (23.0) | 267 (17.3) |
| Depression | 126 (12.8) | 138 (24.6) | 264 (17.1) |
| Arthritis | 461 (46.8) | 218 (38.9) | 679 (43.9) |
